# Influence of Sex on Basal and Dickkopf-1 Regulated Gene Expression in the Bovine Morula

**DOI:** 10.1371/journal.pone.0133587

**Published:** 2015-07-21

**Authors:** Anna C. Denicol, Beatriz C. S. Leão, Kyle B. Dobbs, Gisele Z. Mingoti, Peter J. Hansen

**Affiliations:** 1 Department of Animal Sciences, D.H. Barron Reproductive and Perinatal Biology Research Program, and Genetics Institute, University of Florida, Gainesville, Florida, United States of America; 2 Laboratory of Physiology of Reproduction, School of Veterinary Medicine, Universidade Estadual Paulista—UNESP, Araçatuba, SP, Brazil; University of Connecticut, UNITED STATES

## Abstract

Sex affects function of the developing mammalian embryo as early as the preimplantation period. There were two goals of the current objective. The first was to determine the degree and nature of differences in gene expression between female and male embryos in the cow at the morula stage of development. The second objective was to determine whether DKK1, a molecule known to alter differentiation of the blastocyst, would affect gene expression differently for female and male morulae. In Experiment 1, female and male embryos were treated with DKK1 at Day 5 after insemination. Morulae were harvested 24 h after treatment, pooled in groups of 20 for microarray analysis and RNA subjected to analysis of gene expression by microarray hybridization. There were 662 differentially expressed genes between females and males and 128 of these genes had a fold change ≥ 1.5 between the two sexes. Of the genes upregulated in females, 49.5% were located in the X chromosome. Functional analysis predicted that cell survival was greater in female embryos. Experiment 2 involved a similar design except that transcripts for 12 genes previously reported to be affected by sex, DKK1 or the interaction were quantified by quantitative polymerase chain reaction. Expression of all genes tested that were affected by sex in experiment 1 was affected in a similar manner in Experiment 2. In contrast, effects of DKK1 on gene expression were largely not repeatable in Experiment 2. The exception was for the Hippo signaling gene *AMOT*, which was inhibited by DKK1. In Experiment 3, embryos produced by fertilization with unsorted sperm were treated with DKK1 at Day 5 and abundance of transcripts for *CDX2*, *GATA6*, and *NANOG* determined at Days 5, 6 and 7 after insemination. There was no effect of DKK1 on expression of any of the three genes. In conclusion, female and male bovine embryos have a different pattern of gene expression as early as the morula stage, and this is due to a large extent to expression of genes in the X chromosomes in females. Differential gene expression between female and male embryos is likely the basis for increased resistance to cell death signals in female embryos and disparity in responses of female and male embryos to changes in the maternal environment.

## Introduction

Sex affects function of the developing mammalian embryo as early as the preimplantation period [1–3]. In the cow, functional differences between female and male embryos include differences in energy metabolism [4], developmental capacity in the presence of high concentrations of glucose [5, 6], amino acid uptake [7], expression of the maternal recognition of pregnancy signal *IFNT* [5] and telomere length [8]. Sexual dimorphism in the preimplantation period is driven by gene expression. About one-third of the genes expressed in the bovine blastocyst show differential expression between females and males [9]. There were 1,667 genes upregulated in females and 2,089 genes upregulated in males [9]. One determinant of sex-dependent gene expression is DNA methylation. In cattle, global DNA methylation was greater for females at the 8-cell stage but greater for males at the blastocyst stage [10]. Sex differences have also been observed for specific differentially-methylated regions [8, 11].

One consequence of the disparity in function of female and male embryos is that developmental programming signals during the preimplantation period cause sexually-dimorphic outcomes later in pregnancy or in the postnatal period [12–17]. For example, female mice born from mothers fed a low protein diet for the first 3.5 d of gestation were heavier at birth and throughout 28 wk of life [13,14] and, as adults, had altered behavioral scores [13], increased blood pressure [14], reduced heart to body weight ratio [13], and increased expression of *Insr* and *Igf1r* in retroperitoneal fat [14]. These variables were not affected in male siblings. Conversely, adult males born from mothers fed a low fat diet during the preimplantation period, but not females, had reduced *Ucp1* expression in retroperitoneal fat [14] and vascular dysfunction [16].

An experiment with bovine embryos is also indicative of sexual dimorphism in response to changes in maternal regulatory signals. Treatment of embryos with CSF2 from Day 5–7 of development caused sex-specific alterations in development later in pregnancy when the conceptus undergoes trophoblast elongation [18]. In particular, embryo length and IFNT accumulation in the uterus at Day 15 was decreased if female embryos were treated earlier with CSF2 while the opposite effect occurred for male embryos.

Bovine embryos can respond to maternal cues as early as the one-cell to four-cell stage [19] and several maternally-derived embryokines can affect the embryo including IGF1 [20], FGF2 [21], CSF2 [18, 22] and DKK1 [23]. It is important to determine whether sexual dimorphism in gene expression exists before the blastocyst stage.

There were two goals of the current objective. The first was to determine the degree and nature of differences in gene expression between female and male embryos at the morula stage of development. To date, sexual dimorphism in global gene expression in bovine embryo has not been determined earlier than the blastocyst stage. The second objective was to determine whether a molecule known to alter development of the bovine embryo would affect gene expression differently for female and male embryos. The molecule tested was DKK1, which is a secreted inhibitor of canonical WNT signaling. Among the tissues in which DKK1 is expressed is the endometrium [24]. Exposure of bovine embryos in vitro to DKK1 from Day 5 to 7 of development increased the percentage of cells committed to the trophectoderm (TE) and hypoblast lineages and increased embryo survival and pregnancy establishment after transfer to recipients [23]. DKK1 also increases the relative number of TE cells in the blastocyst of the pig [25]. A finding that DKK1 affects gene expression differently for female than male embryos would imply that long-term effects of the WNT antagonist could be dependent on embryo sex.

## Materials and Methods

### Embryo Production

Cumulus-oocyte complexes were retrieved by slicing follicles ~ 3 to 8 mm in diameter from bovine ovaries obtained from a local abattoir. Embryos were produced from matured cumulus-oocyte complexes using procedures previously described [10]. X- and Y-sorted sperm from five bulls from different *Bos taurus* breeds was obtained from Genex Cooperative, Inc. (Shawano, WI, USA). Each fertilization procedure used separate pools of X and Y-sorted sperm from three randomly-selected bulls in a total of 9 procedures. For each procedure, the same three bulls provided X and Y-sorted sperm (note, sexed semen results in about 90% of resultant offspring of the desired sex) After thawing, sperm were purified using ISolate Separation Medium (Irvine Scientific, Santa Ana, CA, USA) and washed with HEPES-SOF [23]. Fertilization of groups of 30 matured cumulus-oocyte complexes was performed in 50 μl drops of SOF-FERT medium covered by mineral oil [26]. Final concentration of sperm in the fertilization drop was 2x10^6^/ml. A solution of PHE [3.5 μl of 1 mM hypotaurine, 2 mM penicillamine, and 250 μM epinephrine in 0.9% (w/v) NaCl] was added to the fertilization drops to stimulate sperm capacitation. Fertilization was carried out for 20–22 h at 38.5°C and a humidified atmosphere of 5% (v/v) CO_2_. At the end of the fertilization period, presumptive zygotes were washed in HEPES-SOF and randomly placed in groups of 25–30 putative zygotes in 45 μl microdrops of SOF-BE1 [21] covered in mineral oil (Sigma-Aldrich, St. Louis, MO, USA). Embryos were cultured at 38.5°C in a humidified atmosphere of 5% (v/v) O_2_, 5% (v/v) CO_2_ and the balance N_2_.

### Experiment 1: Effect of Sex and DKK1 Treatment at the Morula Stage on Global Gene Expression

#### Treatment with DKK1

On Day 5 of development, embryos were treated by addition of 5 μl of vehicle [Dulbecco’s phosphate buffered saline (DPBS) containing 0.1% (w/v) bovine serum albumin (BSA)] or 1000 ng/ml recombinant human DKK1 (to achieve a final concentration of 100 ng/ml; R & D Systems, Minneapolis, MN, USA) to each 45-μl drop. Bovine and human DKK1 share 90% identity based on the Basic Local Alignment Tool (BLAST) and recombinant human DKK1 has been shown to affect cell differentiation in bovine blastocysts [23]. Pools of 20 morulae (embryos > 16 cells) were harvested 24 h after treatment (Day 6 of development) in five replicates. A total of 2,945 presumptive zygotes were used in the experiment. Morulae were defined as embryos that were not blastocysts (i.e., possessing a blastocoel) and had > 16 cells (i.e., too many cells to count visually). Morulae included both compacted and non-compacted embryos. No blastocysts were included in any pool.

Harvested morulae were washed twice in diethylpyrocarbonate (DEPC)-treated DPBS containing polyvinylpyrrolidone 0.1% (w/v) (DPBS/PVP 0.1%) and incubated with 0.1% (w/v) protease from *Streptomyces griseus* (Roche, Nutley, NJ, USA) in DEPC-treated DPBS/PVP for removal of the zona pellucida, followed by three more washes in DEPC-treated DPBS/PVP. Morulae were then placed in 5 μl of DEPC-treated DPBS/PVP in microcentrifuge tubes, snap frozen in liquid nitrogen and stored at -80°C until further analysis.

Data on embryonic development were analyzed by analysis of variance using the Generalized Linear Model of SAS (SAS Institute, Cary, NC, version 9.3). The experimental design involved a 2 x 2 factorial arrangement of treatments and included sex, treatment and the interaction as fixed effects and replicate as a random effect.

#### RNA extraction and microarray analysis

RNA was extracted from five pools of 20 morulae of each sex and treatment using the Qiagen RNeasy micro kit (Valencia, CA, USA) following the manufacturer’s instructions. The RNA isolation procedure included DNase treatment. Elution was performed in a volume of 9 μl, with the first eluate being applied to the column a second time to increase RNA yield. RNA concentration and quality were assessed using Agilent 2100 Bioanalyzer (Santa Clara, CA, USA); RNA integrity number ranged from 7.8 to 10.0. Due to low starting RNA concentration (ranging from 100 to 900 pg/μl), RNA was amplified using Ovation Pico WTA System V2 kit (Nugen, San Carlos, CA, USA) according to manufacturer’s instructions. The Ovation system uses single primer isothermal amplification technology to reverse-transcribe RNA and linearly amplify cDNA from starting amounts as low as 500 pg of total RNA. For synthesis of the first cDNA strand, primer annealing was allowed by incubation of samples and reagents for 2 min at 65°C, followed by first strand synthesis for 2 min at 4°C, 30 min at 25°C, 15 min at 42°C, and 15 min at 70°C. Synthesis of the second cDNA strand was accomplished by incubation for 1 min at 4°C, 10 min at 25°C, 30 min at 50°C, and 20 min at 80°C; cDNA purification was achieved through magnetic sorting using Agencourt RNAClean XP beads (Ovation Pico WTA System V2 kit, Nugen) and single primer isothermal amplification was accomplished by incubation for 1 min at 4°C, 75 min at 47°C, and 5 min at 95°C.

Five micrograms of amplified cDNA were fragmented to sequences of 50 to 100 bases long and labeled using Encore Biotin Module (Nugen). Labeling consisted of addition of a biotin-labeled nucleotide at the 3’ end of the fragmented cDNA. Fragmented and labeled amplified cDNA was then added to the hybridization cocktail (Affymetrix, Santa Clara, CA, USA) in a concentration of 23 ng/μl. Hybridization mix consisted of 1.15 μg of target cDNA, 1x hybridization buffer, 10% dimethyl sulfoxide, 55 pM control oligonucleotide B2, 1x eukaryotic hybridization controls (bioB, bioC, bioD, cre) and water to complete a volume of 200 μl. Arrays used were the Affymetrix Bovine Gene 1.0 ST and hybridization conditions were 18 h at 45°C. Washing and staining were performed using fluidics protocol FS450_007 according to instructions provided by the manufacturer. Arrays were scanned using the GeneChip Scanner 3000 7G (Affymetrix). All procedures from RNA quality analysis, amplification and reverse transcription of RNA to array scanning were performed at the Gene Expression Core Laboratory of the Interdisciplinary Center for Biotechnology Research of the University of Florida. Raw transcriptome data can be accessed from Gene Expression Omnibus using accession number GSE 60098.

#### Preprocessing and analysis of microarray data

Image files were uploaded to Affymetrix Gene Expression Console software for quality control analysis. The robust multi-array average method was used for background correction, log2 transformation and quantile normalization of data. Statistical analysis of differentially expressed genes was carried out using JMP Genomics (SAS Institute Inc., Cary, NC, USA). Data were analyzed by ANOVA and the model included effects of treatment, sex, and the interaction. Genes were considered to be expressed when mean signal intensity was one standard deviation above the mean intensity for negative control probes; this criterion resulted in identification of expression of 9,933 transcripts. Fold change was calculated as 2^*x*^, where *x* is the difference in least-squares mean intensity for that gene between comparisons of interest. Sequential goodness of fit test [27] was used as a multiple comparison correction test.

Three different sets of criteria were used to define differentially expressed genes (DEG) as affected by embryo sex, DKK1 treatment and the interaction between treatment and sex. The criterion for the first set was genes where there was an effect of P < 0.05. The second was those genes where the effect was P < 0.05 and the fold change was ≥ 1.5 or < 0.66 (or for interactions, treatment was ≥ 1.5 fold or < 0.66 fold in females, males or both sexes). The third set of criteria was genes where the effect was associated with P < 0.05 and the gene was retained after correcting with the sequential goodness-of-fit test.

The sets of DEG as defined by the criteria of P < 0.05 and fold change ≥ 1.5 or < 0.66 were uploaded to Ingenuity Pathway Analysis (IPA, Qiagen) software for clustering of genes into common biological, cellular and molecular functions predicted to be changed as a result of differential gene expression. Functions were considered significant when P < 0.05. The upstream analysis function of IPA was used to predict molecules that could be responsible for regulation of DEGs. Transcription regulators, cytokines, enzymes, G-protein coupled receptors, ligand-dependent nuclear receptors, transmembrane receptor, chemical–endogenous mammalian, and growth factors regulating at least 3 DEG and associated with significant p-values are described.

Chi-square tests were performed to analyze if distribution of chromosomal location of DEG for female and male embryos differed from the expected frequency for each chromosome. The analysis was done for all DEG (X-chromosome only) and for those genes in which the P < 0.05 and fold-change was > 1.5 or < 0.66 (all chromosomes). The expected frequency was calculated by determining the percent of the 9,933 expressed genes that were located on each chromosome.

### Experiment 2: Confirmation of Effects of Sex and DKK1 on Gene Expression as Determined by qPCR

An independent experiment was conducted to verify effects of sex, DKK1, and the interaction on gene expression. Female and male embryos were cultured as described earlier, treated with 100 ng/ml DKK1 or vehicle on Day 5, and morulae harvested 24 h later. RNA extraction and isolation from pools of 20 morulae (n = 4 per treatment for most genes and 8 per treatment for *AMOT* and *PRDM*) followed the same procedure described earlier.

Transcript abundance was measured by qPCR for 12 genes that were affected in the previous experiment by sex, treatment, or treatment x sex (*AMOT*, *APOA1*, *DDX3Y*, *FKBP11*, *PRDM4*, *RPP38*, *SEC22B*, *SMAD3*, *TUBB2B*, *XIAP*, *XIST* and *WLS*) as well as three reference genes (*GAPDH*, *SDHA* and *YWHAZ*). Primers for each gene (S1 Table) were designed using PrimerQuest (IDT DNA, Coralville, IA, USA), except for primers that were used previously as described for *GAPDH* [28], *SDHA* [29], *YWHAZ* [29] and *XIST* [30]. Primers were validated using pools of cDNA from 40 blastocysts. Validation included generation of a standard curve of at least three points with a two-factor dilution between two subsequent points. Curve slopes were between -3.02 and -3.20. Amplicons were submitted for sequencing and matching to the intended gene was verified using the Basic Local Alignment Search Tool feature (National Center for Biotechnology Information).

The protocol for qPCR was as follows. Reverse transcription was performed with the High Capacity cDNA Reverse Transcription Kit (Applied Biosystems, Carlsbad, CA, USA) following instructions of the manufacturer. Samples of cDNA were incubated with master mix containing random hexamer primers, nucleotides, and reverse transcriptase for 10 min at 25°C, 120 min at 37°C, and 5 min at 85°C. Negative controls were obtained by subjecting samples to the same protocol without reverse transcriptase. The cDNA was stored at -20°C until further use. Quantitative real-time polymerase chain reaction (qPCR) was performed with CFX96 Real-Time PCR detection System and SsoFast EvaGreen Supermix with Low ROX (Bio-Rad, Hercules, CA, USA). Each reaction consisted of 1 μl forward primer (0.5 μM), 1 μl reverse primer (0.5 μM), 10 μl SsoFast EvaGreen Supermix with Low Rox (Bio-Rad), 6.8 μl 0.1% (v/v) diethylpyrocarbonate-treated H_2_O and 1.2 μl cDNA sample. The PCR protocol consisted of denaturation at 95°C for 30 sec followed by 40 cycles of 95°C for 5 sec and 60°C for 5 sec. Melt curve analysis consisted of one cycle of 65°C to 95°C with 0.5°C increments every 5 sec.

The cycle threshold (C_t_) for each gene of interest was normalized to the geometric mean of three reference genes (*GAPDH*, *SDHA* and *YWHAZ*) to generate ΔC_t_ values that were used for statistical analysis by analysis of variance using the Proc GLM procedure of SAS. The model included effects of sex, treatment and the interaction or, for *AMOT* and *PRDM*, sex, treatment, replicate and all interactions. Average fold changes were calculated based on ΔΔC_t_ values (least-squares mean for the ΔC_t_ of the gene in the effect of interest minus least-squares mean for the ΔC_t_ of the same gene from the reference category).

### Experiment 3: Effects of DKK1 on Expression of Pluripotency and Differentiation Genes During the Morula to Blastocyst Stages

Effects of DKK1 on expression of *CDX2*, *GATA6*, and *NANOG* for embryos produced with conventional (i.e., non-sorted semen) were evaluated. Embryos were produced as described before except that fertilization was performed using a pool of non-sorted spermatozoa from 3 bulls. The experiment was replicated four times and a different pool of 3 bulls was used for each replicate. Embryos were treated with either 100 ng/ml DKKI or vehicle beginning at Day 5 of development. Embryos were collected either immediately before treatment (Day 5, all embryos > 16 cells), at Day 6 (24 h after treatment; all embryos > 16 cells and before the early blastocyst stage) or at Day 7 (48 h after treatment, blastocysts). Harvested embryos were placed into pools of 20 (Day 7) or 30 (Days 5 and 6), exposed to 0.1% (w/v) proteinase from *Streptomyces griseus* for removal of the zona pellucida and stored at -80°C until analysis by qPCR. RNA extraction and isolation was performed using the PicoPure RNA isolation kit (Applied Biosystems, Carlsbad, CA, USA). Analysis of total RNA concentration and quality was performed with a Nanodrop analyzer (Thermo Scientific, Waltham, MA, USA), followed by treatment with 2 U DNase (New England Biolabs, Ipswich, MA, USA). DNAse-treated samples were reverse transcribed using the High Capacity cDNA Reverse Transcription Kit (Applied Biosystems). Negative controls were obtained by incubation of the same samples without reverse transcriptase. The cDNA was stored at -20°C until further use.

Four pools of embryos were used for expression analysis by qPCR using procedures similar to those described above. Transcript abundance was measured for *CDX2*, *GATA6*, and *NANOG* as well as for three reference genes: *GAPDH*, *YWHAZ* and *SDHA*. Primers for *GATA6* [31] and *NANOG* [32] were described previously. Other primers were the same as for Experiment 2. Primer sequences are described in S1 Table. All primers were validated as described for Experiment 2.

Following qPCR, the geometric mean of the C_t_ values for the reference genes was calculated, and this value was used to calculate the ΔC_t_ (subtraction of geometric mean of C_t_ of reference genes from the C_t_ of genes of interest). Statistical analysis was based on ΔC_t_ values. Data were analyzed by the Proc GLM procedure of SAS using a model with effects of sex, treatment, replicate and all interactions. For data presentation, ΔΔC_t_ was calculated by subtracting the mean ΔC_t_ of control and DKK1-treated embryos from ΔC_t_ values from the control at Day 5. Fold change was calculated by the formula 2^-ΔΔ Ct^.

## Results

### Experiment 1: Effect of Sex and DKK1 Treatment at the Morula Stage on Global Gene Expression

#### Cleavage and development to the morula stage

Cleavage rate at Day 3 of development was 48.7 ± 0.8% for female embryos and 44.6 ± 0.8% for male embryos (least-squares means ± SEM). Following treatment at Day 5, development of female embryos to the morula stage at Day 6 was 22.1 ± 1.3% for control and 23.1 ± 1.3% for DKK1. Values for male embryos were 20.0 ± 1.3% for control and 19.0 ± 1.3% for DKK1. Of embryos that cleaved, the percent of female embryos that became morula were 43.4 ± 2.6% for control and 46.3 ± 2.5% for DKK1. Values for male embryos were 42.9% ± 2.5% and 41.7± 2.5% for control and DKK1, respectively. There were no significant differences in any of the above-mentioned variables.

#### Numbers of differentially expressed genes identified by microarray

A total of 9,933 transcripts were identified as being expressed. A summary of the total number of DEG is shown in Table 1. There were 662 genes whose expression differed between female and male embryos (368 upregulated in females and 294 in males), with 128 having a fold change > 1.5 or < 0.66 (94 upregulated in females and 34 upregulated in males). Using sequential goodness-of-fit as a correction for multiple testing, 135 genes varied with sex. There were 367 genes affected by the main effect of DKK1 of which 38 had a fold change ≥had a fold changNone of the effects of DKK1 were significant after correcting for multiple testing. There were 437 genes affected by the DKK1 x sex interaction (i.e., DKK1 had a different effect in females and males). Of these, 159 experienced a fold difference in females or males ≥experienced a f and 50 were significant after correction for multiple testing. A list of DEG is available in S1 File.

**Table 1:** Number of differentially expressed genes due to DKK1, sex and the interaction.

	Number of differentially expressed genes		
Statistical analysis used	DKK1	Sex	DKK1 x sex
P < 0.05	367	662	437
P < 0.05 and fold change ≥ 1.5	38	128	159
Sequential goodness-of-fit test	0	135	50

#### Physical location of genes differentially expressed between female and male embryos

**Fig 1:**
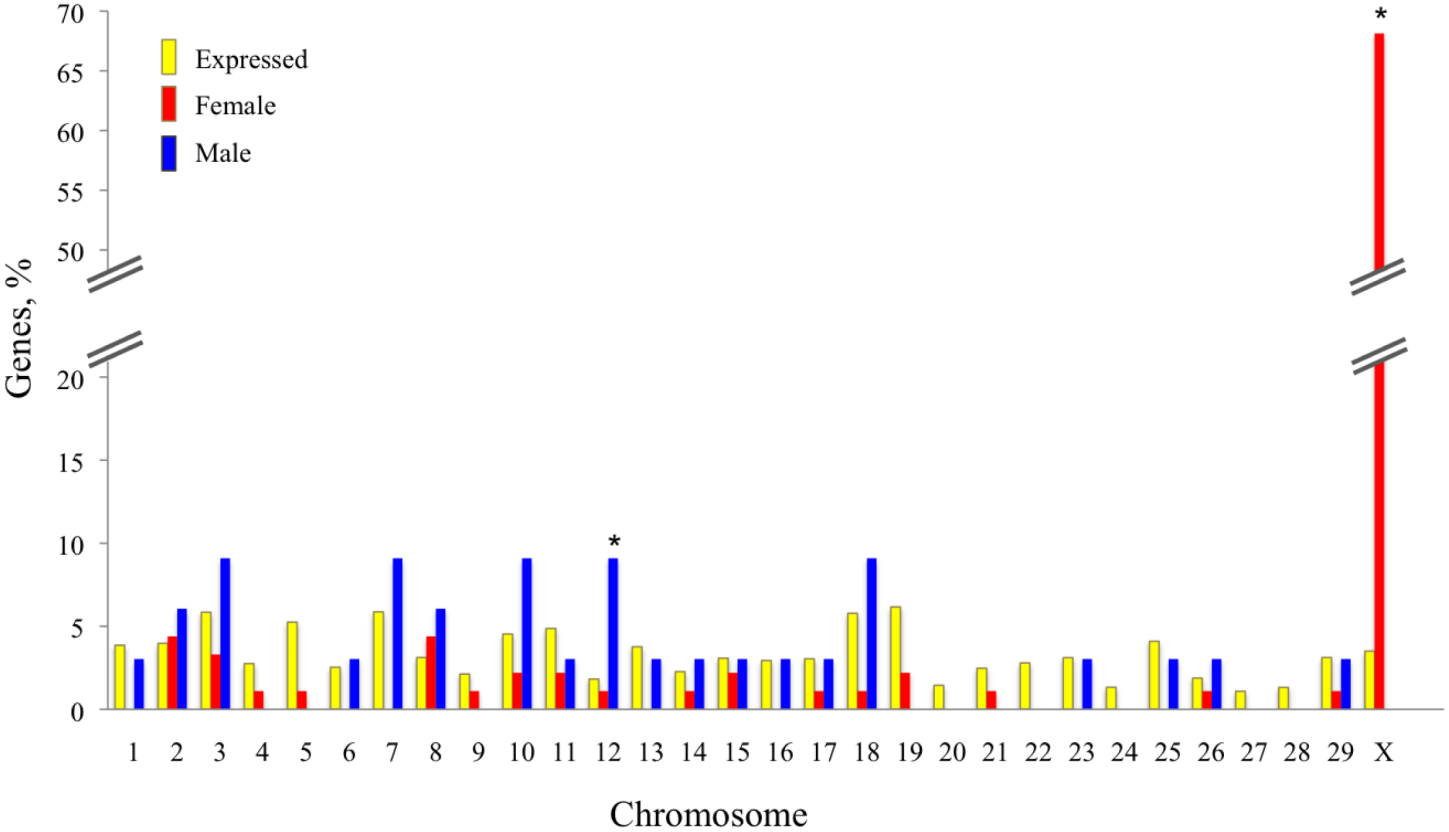
Chromosomal location of genes differentially expressed between female and male embryos. Yellow bars represent the percent of the 9,933 expressed genes located on each chromosome. The red bars represent the percent of genes upregulated in female embryos (those P < 0.05 and ≥ 1.5 fold) that are located on each chromosome while the blue bars represent the percent of genes upregulated in male embryos (P < 0.05 and ≥ 1.5 fold) that are located on each chromosome. Asterisks represent chromosomes in which the frequency of upregulated genes is higher than expected due to chance based on overall distribution of expressed genes on each chromosome (**, P<0.01; ***, P<0.001).

Of the 368 genes whose expression was upregulated in female embryos, 182 (49.5%) were on the X-chromosome. This represents 52.8% of the 345 X-lined genes that were detected as being expressed by the morula. Frequency of upregulated genes on the X chromosome was higher than expected due to chance by chi-square analysis (P < 0.001). Of the 294 genes whose expression was upregulated in male embryos, 3 (1.0%; *DDX3Y*, *EIF1AY*, and *UTY*) were on the Y chromosome. Array design does not allow calculation of the frequency of expressed genes located on the Y chromosome so it was not possible to analyze whether frequency of expression for upregulated genes on the Y chromosome deviated from expected. When only the genes in which sexual dimorphism was greatest were considered, those with P < 0.05 and i 1.5 or < 0.66 fold, 65 of the 94 genes upregulated in females were on the X chromosome (69.1%) and 3 of the 34 genes upregulated in males were on the Y chromosome (8.8%). Frequency of upregulated genes on the X chromosome was higher than expected due to chance by chi-square analysis (P < 0.001). No other chromosome had a frequency of genes upregulated in females at a higher frequency than expected due to chance (Fig 1). There was an overrepresentation of genes upregulated in male embryos on chromosome 12 (P < 0.01) (Fig 1).

#### Predicted molecular and cellular functions affected by sex

**Fig 2:**
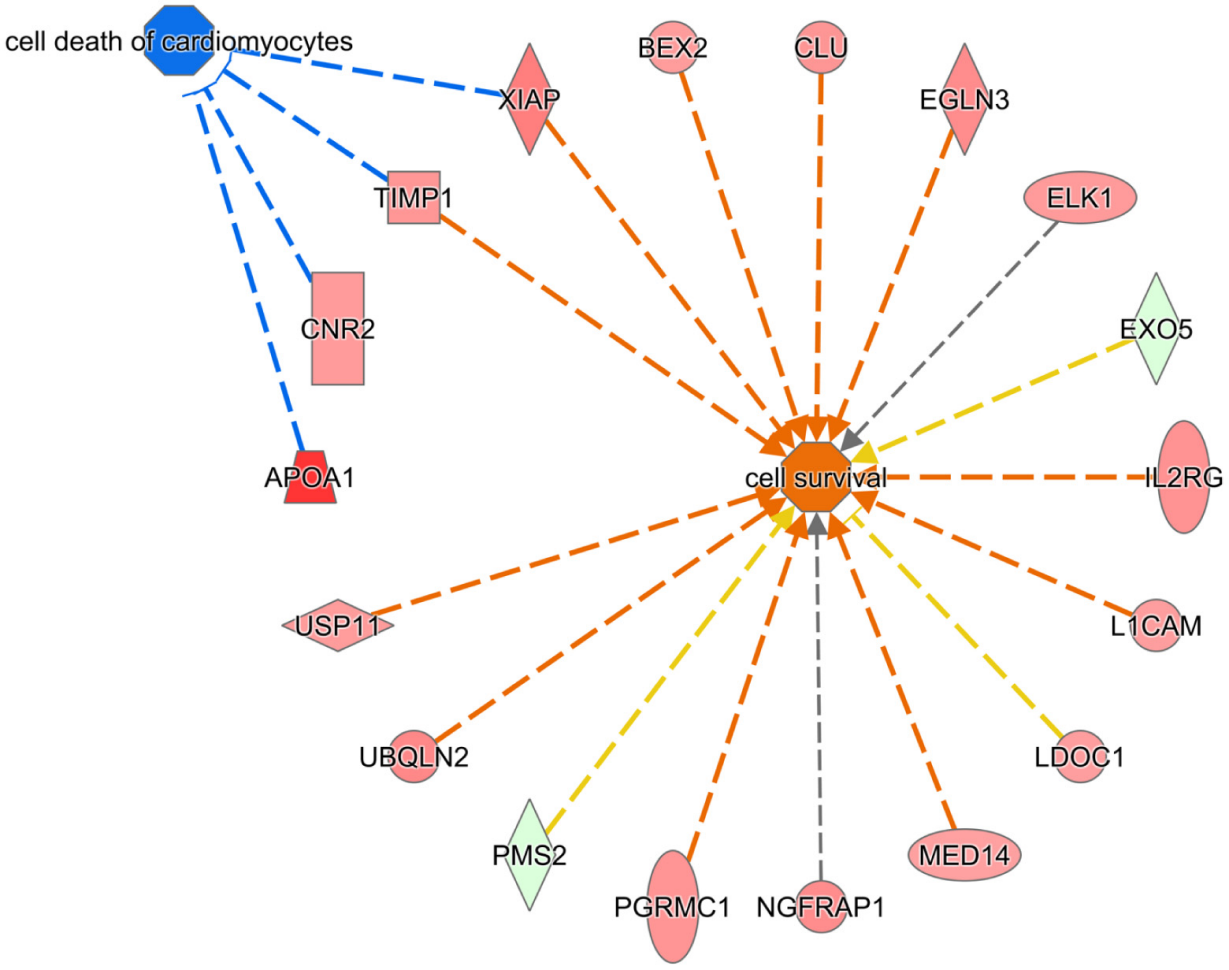
Differences in gene expression between female and male embryos is predictive that cell survival is enhanced in females. Genes upregulated in females are in red while genes upregulated in males are in green. Cellular and molecular functions predicted to be activated are in orange while functions predicted to be inhibited in blue. Orange arrows represents predicted activation of the function, blue arrows represent predicted inhibition, yellow arrows indicates that the relationship is inconsistent with the prediction while gray lines represent that the effect is not predicted. Note *EXO5* is identified as DEM1 in the microarray database.

Using IPA, 79 of the 94 genes upregulated in female embryos and 32 of the 34 genes upregulated in male embryos were mapped to known functions. The five most-significant molecular and cellular functions represented in the list of DEG included cell death and survival (n = 29 genes), cellular movement (16 genes), cell morphology (16 genes), cellular assembly and organization (14 genes) and DNA replication, recombination and repair (11 genes). Changes in gene expression indicated that cell survival was increased in female embryos as compared to males (Fig 2). Of the 16 DEG upregulated in females that are involved in cell survival, 12 are located on the X chromosome.

Fourteen genes were associated with embryo development. Of these, 12 were upregulated in females (*AP1S2*, *ARAF*, *CNR2*, *DMD*, *EGLN3*, *ELK1*, *IL13RA1*, *IL2RG*, *L1CAM*, *RIMS3*, *RPS6KA3*, and *TIMP1*) and 2 were upregulated in males (*RPB4* and *ZBTB5*). Nine of the 12 genes upregulated in females were located on the X chromosome. The exceptions were for *CNR2*, *EGLN3* and *RIMS3*.

IPA was used to identify putative regulators of sex-dependent gene expression (S1 Fig). Five regulators were predicted (P<0.05) to increase gene expression in females. These were estradiol-17β (14 genes), TNFA (12 genes), TGFB1 (9 genes), and CD40LG (7 genes). One regulator, the hyaluronidase MGEA5, was predicted to be inhibited in females because 5 genes inhibited by MGEA5 were increased in females.

#### Effects of DKK1 on expression of genes involved in blastocyst differentiation and pluripotency

**M**icroarray data were examined for effects of DKK1 on genes involved in TE and hypoblast formation (Table 2). Two genes implicated in TE formation were not expressed in the morula, *ELF5* and *EOMES*, and two others, *TCFAP2C* and *YAP1*, were not on the array. None of the other 8 genes involved in TE formation were significantly affected by treatment. However, *AMOT* expression tended to be reduced by DKK1 (P = 0.091). The effect of DKK1 only occurred in male embryos (expression was 2-fold higher in controls compared to DKK1 embryos) and there was a DKK1 x sex interaction (P = 0.012). Of two genes involved in hypoblast formation, *FGF4* and *FGFR2*, both were expressed. There was no effect of sex, DKK1 or the interaction on expression of *FGF4*. Expression of FGFR2 was not affected by sex or the interaction but was 1.28-fold higher for DKK1-treated embryos than control embryos (P = 0.016). Both of two genes involved in pluripotency of the inner cell mass (ICM), *NANOG* and *POU5F1*, were expressed but neither was affected by sex, DKK1 or the interaction.

**Table 2:** Effects of sex, DKK1 and the interaction on expression of genes involved in blastocyst differentiation and pluripotency.^a^

	Female		Male		P values		
Gene	Control	DKK1	Control	DKK1	Sex	DKK1	DKK1 x sex
*AMOT*	6.581	6.819	7.128	6.075	0.671	0.091	0.012
*CDX2*	7.546	7.778	7.645	7.357	0.646	0.935	0.460
*ETS2*	5.052	4.816	4.729	5.026	0.821	0.820	0.293
*FGFR2*	4.697	5.149	4.724	4.989	0.626	0.016	0.604
*FGF4*	4.856	5.224	5.474	4.993	0.694	0.908	0.392
*GATA3*	7.912	8.173	8.099	8.267	0.506	0.314	0.824
*GATA6*	10.549	10.681	10.548	10.494	0.219	0.603	0.226
*KLF5*	8.571	8.581	8.488	8.708	0.821	0.241	0.284
*LATS1*	7.393	7.480	7.362	7.778	0.553	0.278	0.470
*LATS2*	5.367	5.301	5.345	5.354	0.849	0.725	0.640
*NANOG*	6.164	6.469	6.894	6.717	0.103	0.823	0.407
*POU5F1*	10.428	10.459	10.465	10.468	0.711	0.787	0.824
*SOX2*	9.393	9.452	9.690	9.514	0.225	0.685	0.421
*TEAD4*	8.571	8.422	8.468	8.514	0.973	0.739	0.526

^a^ Units are log2 of fluorescent intensity.

### Experiment 2: Confirmation of Effects of Sex and DKK1 on Gene Expression as Determined by qPCR

An independent experiment using distinct pools of embryos was conducted to confirm effects of sex, DKK1, and DKK1 x sex on gene expression by qPCR. Genes selected for evaluation included those that were upregulated in female (*APOA1*, *XIAP* and *XIST*) or male embryos (*DDX3Y*), those affected by the main effect of DKK1 (*FKBP11*, *SEC22B* and *TUBB2B*), and genes in which there was a DKK1 x sex interaction (*AMOT*, *PRDM4*, *RPP38*, *SMAD3* and *WLS*). As shown in Table 3, microarray results were confirmed by qPCR with statistical significance for the four genes affected by sex (*APOA1*, *DDX3Y*, *XIAP*, and *XIST*). In contrast, none of the genes affected by the main effect of DKK1 in the microarray experiment exhibited a similar significant pattern in the independent experiment. There were no effects of DKK1 on expression of *SEC22B* and *TUBB2B* and expression of *FKBP11* was decreased by DKK1 in contrast to the microarray experiment in which gene expression was increased. Of the five genes affected by a DKK1 x sex interaction by microarray, there were no significant interactions identified in the independent experiment. Thus, results confirm the conclusion from the microarray experiment that sex alters the transcriptome of the bovine morula but few genes are affected by DKK1 or the interaction between DKK1 and sex.

**Table 3:** Comparison of the fold-change in gene expression due to sex, DKK1 and DKK1 x sex as determined by microarray and by qPCR. ^a^

Gene	Microarray (Experiment 1)			qPCR (Experiment 2)		
Sex:	Fold change fem vs male		P-value	Fold change fem vs male		P-value
*APOA1*	3.25		< 0.01	2.5		< 0.010
*DDX3Y*	0.11		< 0.01	0.11		< 0.010
*XIAP*	2.05		< 0.01	1.55		< 0.05
*XIST*	3.27		< 0.01	3.76		< 0.01
DKK1:	Fold change DKK1 vs control		P-value	Fold change DKK1 vs control		P-value
*FKBP11*	1.82		0.02	0.84		0.04
*SEC22B*	1.81		0.03	0.80		0.31
*TUBB2B*	0.226		0.01	1.02		0.95
Interaction:	Fem DKK1 vs fem control	Male DKK1 vs male control	P-value	Fem DKK1 x fem control	Male DKK1 x male control	P-value
*AMOT*	1.179	0.482	0.012	0.86	0.76	0.58
*PRDM4*	1.074	0.504	0.005	0.92	0.80	0.39
*RPP38*	0.460	1.541	0.005	0.79	0.80	0.98
*SMAD3*	1.849	0.646	0.003	0.77	0.95	0.28
*WLS*	1.576	0.541	0.005	1.28	1.27	0.98

^a^ Abbreviation: fem—female.

**Fig 3:**
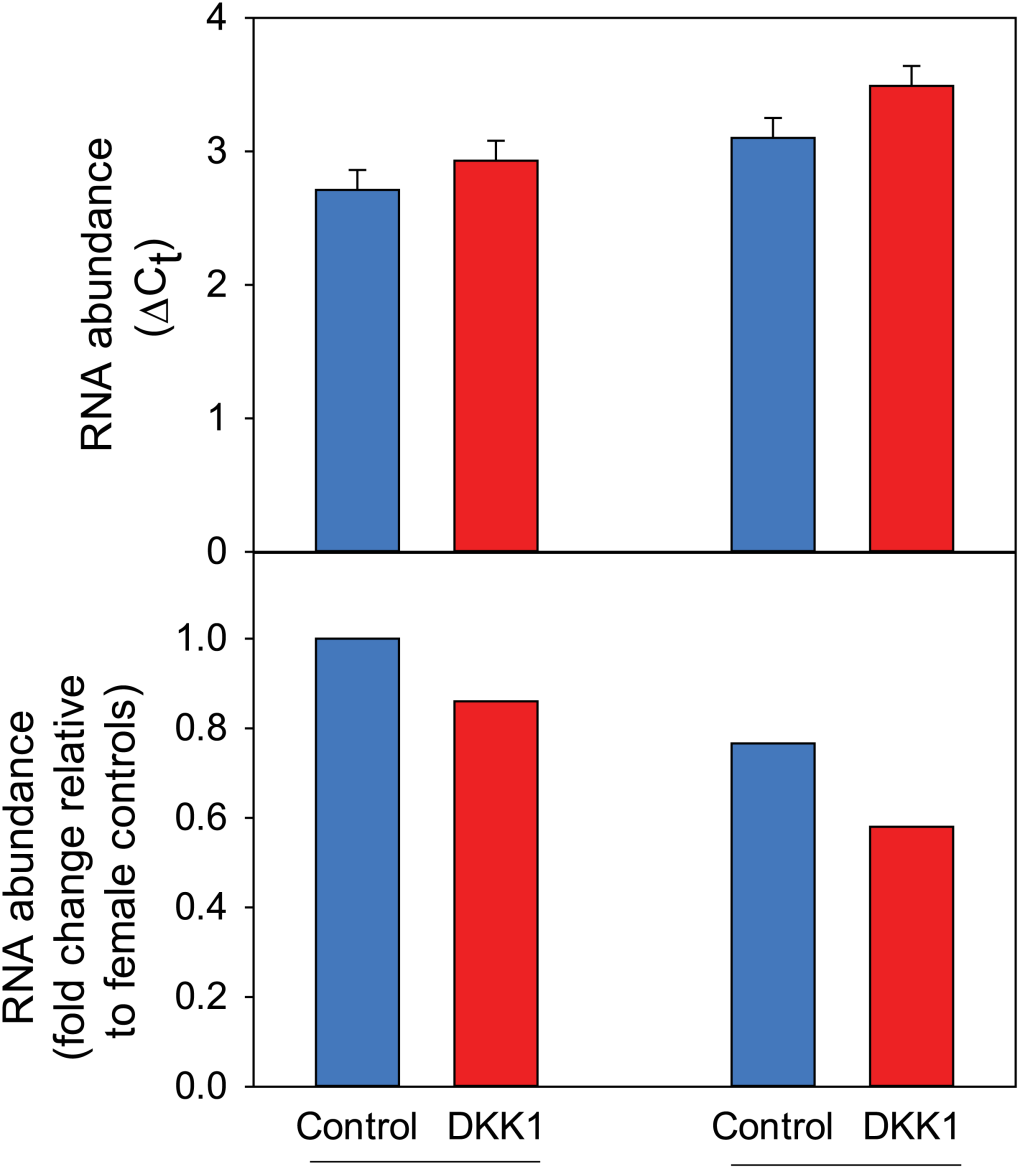
Effect of sex and DKK1 on AMOT expression in Day 6 morula (Experiment 2). Data are shown as ΔC_t_ data (top panel) and as fold-change relative to female controls (bottom panel). Data are least-squares means ± SEM (top) or were calculated from the least-squares means of ΔC_t_ (bottom). RNA abundance was affected by sex (P = 0.0527) and DKK1 ((P = 0.0222) but not by the interaction (P = 0.5791).

In the microarray experiment, expression of *AMOT* tended to be affected by DKK1 (P = 0.091) and was affected by the sex x DKK1 interaction (P = 0.012). The interaction was not significant in the confirmation experiment but there was an overall effect of DKK1 on expression of *AMOT* (P = 0.0222). In particular, DKK1 decreased *AMOT* expression in both female and male embryos (Fig 3). Expression was higher for females (P = 0.0527) but there was no interaction between DKK1 x sex (P = 0.5791).

### Experiment 3: Effects of DKK1 on Expression of Pluripotency and Differentiation Genes During the Morula to Blastocysts Stages

**Fig 4:**
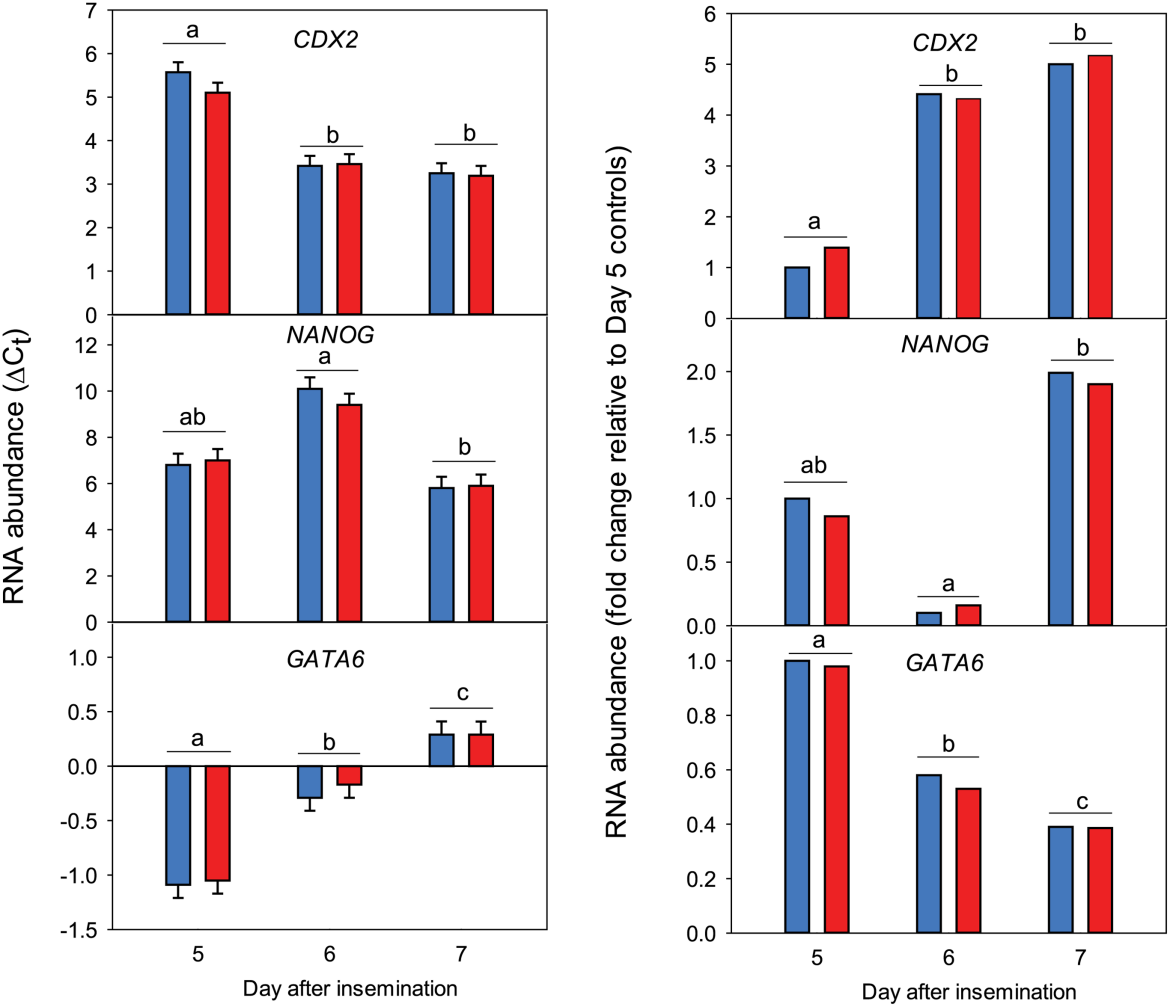
Effect of day after insemination on expression of *CDX2*, *NANOG* and *GATA6* (Experiment 3). Data are shown as ΔC_t_ data (left panel) and as fold-change relative to Day 5 controls (right panel). Data are least-squares means ± SEM (left) or were calculated from the least-squares means of ΔC_t_ (right). Embryos were morulae (Days 5 and 6) or blastocysts (Day 7). Day of insemination is considered Day 0. Blue bars represent control and red bars represent DKK1. Day effects are indicated by superscripts. Days with different superscripts differ (P<0.05). There were no effects of DKK1 or day x DKK1.

Results are shown in Fig 4. There were day effects for *CDX2* (Day 5 vs Day 6 + Day 7, P = 0.0464), *NANOG* (P = 0.0499) and *GATA6* (P = 0.003). Expression of *CDX2* was higher on Days 6 and 7 than on Day 5, *NANOG* increased at Day 7 from a nadir at Day 6 and *GATA6* decreased from Day 5 to 7. There were no effects of DKK1 or day x DKK1 on expression of any of the genes.

## Discussion

Results reported here demonstrate that female and male bovine embryos have different patterns of gene expression as early as the morula stage. The differences in gene expression are likely to cause sex-dependent variation in cell survival and in regulation of pluripotency and differentiation of the early embryo. There was no consistent evidence that sex affected regulation of gene expression by the WNT signaling antagonist DKK1. In fact, this molecule, which can alter proportion of cells in the blastocyst that are TE, ICM and hypoblast [23, 25] had little effect on transcription at the morula stage. Thus, it is likely that actions of DKK1 on lineage commitment in the blastocyst stage are dependent upon post-transcriptional modifications [33] or differences in gene expression later in development. One gene regulated by DKK1 that may lead to downstream effects on expression of genes involved in TE formation was the Hippo signaling molecule *AMOT*.

Comparison of the characteristics of sexual dimorphism in gene expression between morula (this study) and blastocysts [9] is indicative that the extent of sexual dimorphism increases from the morula to the blastocyst stage. The total number of expressed transcripts in Day 6 morulae was 9,933. This value is similar to values of 9,322–9,489 expressed genes in blastocysts [9,34]. In morula, expression of a total of 662 genes (7.1% of the 9,933 expressed genes) was affected by sex before correction for multiple testing. By comparison, 40.2% of genes expressed in the blastocyst exhibited differences in expression between female and male embryos [9]. In contrast to the situation for the total number of DEG affected by sex, morulae and blastocysts were similar in terms of genes whose expression was most affected by sex. In the morula, there were 94 genes upregulated in females ≥ 1.5-fold and 34 genes upregulated in males ≥ 1.5 fold. This is similar to the blastocyst where there were 95 genes upregulated in females ≥ 1.5 fold and 12 in male [9].

Differences in gene expression between female and male embryos can be largely ascribed to differential expression of genes on the X chromosome. Of the 368 genes whose expression was upregulated in female embryos, 182 (49.5%) are on the X-chromosome. For those 94 genes whose expression was ≥ 1.5 for females than males, 69.1% are on the X chromosome (69.1%). Similar results were observed for blastocysts [9]. The preponderance of DEG on the X chromosome is not surprising because X chromosome inactivation is incomplete at the morula and blastocyst stages in the cow. Expression of the X inactivating gene *XIST* starts at the 8-cell stage [35]. Late replication of one X chromosome is detected in some early blastocysts and is probably complete at some point between then and Day 14–15 of gestation [35, 36]. It is likely that some X-chromosome inactivation has occurred by the morula stage because, like for the blastocyst [3], most the DEG overexpressed in females exhibited a fold change less than 2. Incomplete X-chromosome inactivation at the blastocyst stage is also a characteristic of development in the rabbit and human while inactivation is complete by the blastocyst stage in the mouse (reviewed by Bermejo-Alvarez et al. [37]).

Expression of genes on the Y-chromosome is also responsible for some of the sex-related transcriptional differences but on a much smaller scale than for genes on the X chromosome. Three Y-linked genes were found to be upregulated in male embryos: *DDX3Y*, *EIF1AY* and *UTY*. These three genes are contained within an 816 kb region of the bovine Y chromosome [38]. *DDX3Y* and *EIF1AY* regulate RNA transcription and translation initiation [39, 40] while *UTY* controls gene expression by promoting demethylation of H3K27 and methylation of H3K4 [41].

Taken together, results are indicative that the morula and blastocyst have a similar number of genes, largely located on the X chromosome, which experience a ≥ 1.5 fold increase in expression. Moreover, the total number of genes differentially regulated between female and male embryos increases as the embryo develops to the blastocyst stage. In the mouse, too, the number of DEG between female and male embryos increases from the 8-cell to blastocyst stages of development [42, 43]. Perhaps the set of genes whose expression is ≥ 1.5 fold higher in females than males early in development drives subsequent changes in gene expression on the X and other chromosomes by the blastocyst stage. Consistent with this idea is the observation that ~50% of the DEG in morula are on the X chromosome versus only 18% in the blastocyst [9].

Sex-dependent differences in gene expression at the morula stage are likely to result in different functional capabilities between female and male embryos. The one molecular function that was predicted to be enhanced in female embryos was cell survival. Most of the genes promoting cell survival in females are on the X chromosome. In addition to the genes identified as regulating cell survival, two other developmental genes upregulated in female embryos, the X-linked gene *ARAF* [44] and the autosomal gene *CNR2* [45–47], prevent cell death in embryonic cells. The idea that female embryos are more resistant to cell death than male embryos is consistent with observation that, in the mouse, female embryos were more resistant to heat stress in vivo or heat shock in vitro than male embryos [48].

Another gene whose expression was higher in female embryos could also potentially alter embryonic development. *ELK1* is a transcription factor targeted by MAPK signaling, which in turn is critical for cell division in early cleavage stages in the mouse [49]. Despite upregulation of *ELK1*, there was no sex effect on development to the morula stage. There are other reports indicating that female and male embryos develop to the blastocyst stage at the same rate [8, 50] but there are more reports that female embryos develop to the blastocyst stage more slowly than males [5,6, 51–53].

One possible reason for reduced development in female embryos is that glucose can be toxic to female embryos at concentrations of 4 mM or above but not to male embryos [52]. However, deviation in sex ratio of in vitro produced blastocysts is observed even when glucose concentrations are too low to be toxic [51, 53]. Reduced development of female embryos has also been ascribed to increased expression of the X-linked gene *G6PD* involved in glucose metabolism to generate NADPH through the pentose phosphate pathway. *G6PD* has been reported to be more highly expressed in female embryos than male embryos at the morula [54] and blastocyst stages [54,55] and inhibition of G6PD has been reported to reduce differences in development between females and males [52]. There was, however, no effect of sex on expression of *G6PD* in the current experiment (S1 File) or in another experiment with bovine blastocysts [9]. Moreover, the gene ontology analyses did not indicate overrepresentation of DEG in pathways associated with glucose metabolism. Perhaps the degree to which *G6PD* is differentially expressed between females and male embryos depends upon culture conditions and female embryos only develop to the blastocyst at a lower rate than males when culture conditions increase expression of *G6PD*.

One consequence of the sexual dimorphism of the preimplantation embryo is that developmental programming signals during this period can lead to different outcomes for females and males [12–17]. In the cow, exposure of Day 5–7 embryos to the maternal regulatory molecule, CSF2, caused sex-specific alterations in development later in pregnancy at Day 15 of gestation [18]. Three of the genes overexpressed in female morulae encode for receptor molecules that could be involved in sex-dependent responses in maternal signals. The autosomal gene *CNR2* encodes for a cannabinoid receptor that has been shown to promote survival of embryonic stem cells and trophoblast stem cells [56]. Two other genes overexpressed in females encode for cytokine receptor subunits. *IL13RA1* encodes for a subunit that forms part of the IL4 and IL13 receptor [57] while *IL2RG* encodes for a signaling component of receptors for IL2, IL4, IL7, IL15, and IL21 [58]. Both of these latter genes are X-linked. The role of interleukins in development of the preimplantation embryo is not well understood. Infusion of mononuclear leukocytes into the uterus of embryo transfer recipients can increase pregnancy rate in cattle [59]. IL4 can increase expression of non-classical major histocompatibility class I molecules on bovine blastocysts [60].

In addition, several of the genes overexpressed in females are regulated by upstream factors that could also be involved in sexual dimorphism in response to maternal cues. One of these, TGFB1, has been reported to promote development of bovine embryos to the blastocyst stage [61, 62]. In contrast, TNFA induces apoptosis in the bovine embryo [63].

One purpose of the experiments described here was to evaluate whether the maternal embryokine DKK1 exerts sex-dependent changes in gene expression in the morula. DKK1 regulates lineage commitment in both cattle [23] and pigs [25]. There were, however, very few genes whose expression was regulated by DKK1 in Experiment 1 and none of the effects of DKK1 were repeatable in Experiment 2. Thus, DKK1 has little effect on gene expression of the bovine morula after 24 h of exposure and an examination of sexual disparity in regulation of gene expression by DKK1 was of limited usefulness.

The lack of large-scale regulation of gene expression by DKK1 is likely to mean that actions of DKK1 on lineage commitment largely involve regulation of genes beyond the time frame examined. Moreover, the increase in relative proportions of TE and hypoblast cells in the blastocyst caused by DKK1 involves actions other than regulation of expression of *CDX2*, required for TE formation, *GATA6*, the transcription factor involved in specification of hypoblast and *NANOG*, required for maintenance of pluripotency in the ICM [64]. This conclusion is based on results of Experiment 3, where DKK1 had no effect on expression of any of these transcription factor genes at days 5, 6 or 7 after insemination.

An important exception to the lack of effect of DKK1 on expression of genes involved in lineage commitment was for *AMOT*. In Experiment, expression of this gene was reduced by DKK1 in male embryos. Further experimentation using qPCR indicated that expression was inhibited by DKK1 in both sexes. Actions of DKK1 on expression of *AMOT* suggest that DKK1 may regulate commitment to TE lineage through inhibition of Hippo signaling. This pathway plays an important role in TE formation in the mouse through regulation of nuclear localization of Yap and resultant Yap- and Tead4-mediated activation of *Cdx2* transcription [65]. The regulatory proteins Lats1/2 and Amot determine nuclear accumulation of Yap; depletion of Amot proteins allows TE-specific nuclear localization of Yap and expression of *Cdx2* at the blastocyst stage [66]. Further research to examine regulation of Hippo signaling by DKK1 may lead to elucidation of the mechanism by which this WNT regulatory protein controls differentiation in the blastocyst.

It may be relevant to effects of DKK1 on hypoblast formation [23] that DKK1 increased expression of *FGFR2* in Experiment 1. Differentiation of ICM to hypoblast in the mouse is driven by actions of Fgf4 mediated by Fgf2r [67] and FGF4 can also cause differentiation of ICM to hypoblast in the cow [68].

In conclusion, experiments presented here indicate that sexual dimorphism in gene expression exists in the bovine preimplantation embryo as early as the morula stage of development. The basis for disparity in gene expression between female and male embryos at this stage is due largely to the presence of two active X chromosomes in the female at this stage of development. There was a preponderance of DEG that were overexpressed in the female and few that were overexpressed in the male. Moreover, half of the genes overexpressed in females are X-linked. Differential gene expression between female and male embryos is likely the basis for increased resistance to cell death signals in female embryos [48]. Evidence for a strong transcriptional regulation at the morula stage by DKK1 was not supported, given that changes in gene expression in both females and males in response to this molecule were modest and could not be confirmed by qPCR. The exception is the decrease in expression of *AMOT* in response to DKK1, which may be an important clue for the role of this molecule as a determinant of cell differentiation in the preimplantation embryo. The topic warrants further investigation. Disparity in responses of female and male embryos to changes in the maternal environment [12–17] is also likely to be dependent on differences in gene expression. Among the genes whose expression is altered by sex that could be involved in this phenomenon are those involved in cannabinoid and interleukin signaling.

## Supporting Information

S1 Fig Predicted upstream regulators of genes differentially expressed between female and male embryos. (PDF)

S1 File Details of effects of sex, DKK1 and the interaction on gene expression. (XLSX)

S1 Table Primers used for qPCR. (PDF)
