# Supplementary material for: Influence of Sex on Basal and Dickkopf-1 Regulated Gene Expression in the Bovine Morula
Source: PLoS One. 2015 Jul 21;10(7):e0133587. doi: 10.1371/journal.pone.0133587 (PMC4510475; doi:10.1371/journal.pone.0133587)
Supplement: S1 Fig — (PDF) [file pone.0133587.s001.pdf]

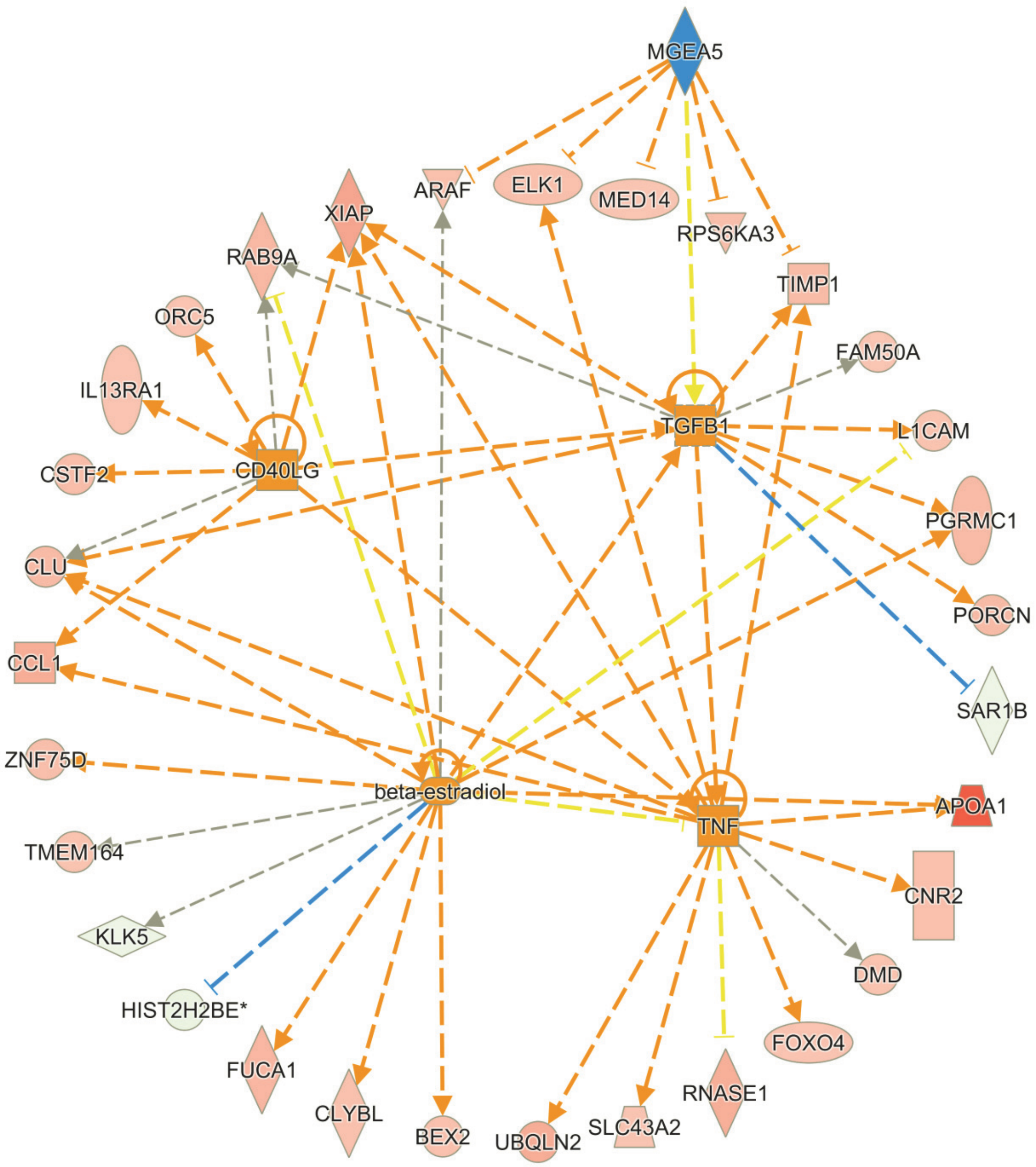

Figure S1. Predicted upstream regulators of genes differentially expressed between female and male embryos. Genes upregulated in females are in red while genes upregulated in males are in green. Putative regulators are in the center: orange symbols for regulators and arrows represent predicted activation while symbols and arrows in blue represent predicted inhibition. Yellow arrows indicate that the relationship is inconsistent with the prediction while gray lines represent that the effect is not predicted. Note HIST2H2BE* is identified as H2B in the microarray database.
