# Supplementary material for: Influence of Sex on Basal and Dickkopf-1 Regulated Gene Expression in the Bovine Morula
Source: PLoS One. 2015 Jul 21;10(7):e0133587. doi: 10.1371/journal.pone.0133587 (PMC4510475; doi:10.1371/journal.pone.0133587)
Supplement: S1 Table — (PDF) [file pone.0133587.s003.pdf]

Table S1. Primers used for qPCR.

Gene	Accession number	Primer sequences
AMOT	NM_001206309.1	F: 5’-CAAGGTCCAGTCCCAGTATTT-3’<br>R: 5’- CTCAGTCCTCATCTTCTGGTT-3’
APOA1	NM_174242.3	F: 5’-CGTGTATGTGGAAGCAATCAAG-3’<br>R: 5’- TGAAACTCCTGGACAACTGG-3’
CDX2	NM_001206299.1	F: 5'-GCCACCATGTACGTGAGCTAC-3'<br>R: 5'-ACATGGTATCCGCCGTAGTC-3'
DDX3Y	NM_001172595.1	F: 5’-CAGTAGAGGTAACCGGCAATA-3’<br>R: 5’-CTTACTCGTTACACACGTCCTAC-3’
FKBP11	NM_001045932.1	F: 5’-GGAGAGAAGCGAAGGGTAATC-3'<br>R: 5’-TTTGATGTGGAACTGATTGCAC-3’
GAPDH	NM_001034034.2	F: 5’-ACCCAGAAGACTGTGGATGG-3’<br>R: 5’- CAACAGACACGTTGGGAGTG-3’
GATA6	DQ126151	F: 5’- ATACTTCCCCCACCACACAA-3’<br>R: 5’- AGCCCGTCTTGACCTGAGTA-3’
NANOG	DQ069776.1	F: 5’-GACACCCTCGACACGGACAC-3’<br>R: 5’-CTTGACCGGGACCGTCTCTT-3’
PRDM4	XM_005206798.1	F: 5’-GACCTGATTGCTGTTCCTTAGA-3’<br>R: 5’- GTATCTGGAGGTCTAACAAGCC-3’
RPP38	NM_001038085.2	F: 5’-CTGGTGAGGATCTGAAGAAAGAA-3’<br>R: 5’-CTATCGGCATCAACGAGGTC-3’
SDHA	NM_174178	F: 5’ GCAGAACCTGATGCTTTGTG-3’<br>R: 5’- CGTAGGAGAGCGTGTGCTT-3’
SEC22B	NM_001076343.2	F: 5’-GTCTCCGTAGCCTGTAAAGATG-3’<br>R: 5’- CTTCAACAGTACCAGAGTCAGG-3’
SMAD3	NM_001205805.1	F: 5’-GCTGACACGGAGGCATATC-3’<br>R: 5’-TGGGAGACTGCACAAAGATG-3’
TUBB2B	NM_001003900.1	F: 5’-CCTCAATCAAGCATGGTCTTTC-3’<br>R: 5’- AATAATGTGGAACTCCTCTGGAA-3’
WLS	NM_001192651.1	F: 5’-ACGTTGGGATTGGAGAGATAAAG-3’<br>R: 5’-TGATGAAGATGCTGGGTGTAAG-3’
XIAP	NM_001205592.1	F: 5’-CTCTGTCGTGAGTTCTGATAGG-3’<br>R: 5’-CATTACATTTGGGACGTGGATG-3’
XIST	NR_001464.2	F: 5'-TTGGCTTTTAGATTAATTTGATGAACAGCAT-3'<br>R: 5'-CCCTTTAGACTAGGCCCATTTCATA-3'
YWHAZ	BM446307	F: 5’- GCATCCCACAGACTATTTCC-3’<br>R: 5’- GCAAAGACAATGACAGACCA-3’
